# Influence of Periodically Varying Slit Widths on Sound Absorption by a Slit Pore Medium

**DOI:** 10.3390/ma18010054

**Published:** 2024-12-26

**Authors:** Keith Attenborough

**Affiliations:** School of Engineering and Innovation, The Open University, Milton Keynes MK7 6AA, UK; keith.attenborough@open.ac.uk

**Keywords:** sound absorption, 3D printing, sinusoidal walls, periodically varying widths

## Abstract

A simple pore microstructure of parallel, identical, and inclined smooth-walled slits in a rigid solid, for which prediction of its geometrical and acoustic properties is straightforward, can yield useful sound absorption. This microstructure should be relatively amenable to 3D printing. Discrepancies between measurements and predictions of normal incidence sound absorption spectra of 3D printed vertical and slanted slit pore samples have been attributed to the rough surfaces of the slit walls and uneven slit cross-sections perpendicular to the printing direction. Theories of the influence of (a) sinusoidal walls and (b) periodically varying uniform slit widths on the normal incidence absorption spectra of a slit pore medium are outlined. Although the slit wall surface and geometrical imperfections due to 3D printing differ from these idealizations, predictions assuming the ideal forms of roughness confirm that pore-wall roughness could account for differences between predictions and data. Pore-wall roughness is predicted to increase both flow resistivity and tortuosity, thereby increasing the low-frequency sound absorption of thin hard-backed layers. The extent to which sinusoidal slit walls or periodically varying uniform slit widths could improve the sound absorption of a low flow resistivity hard-backed layer containing identical vertical slits is explored.

## 1. Introduction

Many designs for creating porous sound absorbers using additive manufacturing involve simple microstructures. These include rigid micro-rods [1], lattices [2], parallel cylinders [3], annular sits [4] and honeycombs [5]. A thin hard-backed rigid solid matrix containing slits is a relatively simple microstructure for which the geometric and acoustic properties can be predicted straightforwardly and, if the slits and separating walls are thin enough, can provide useful sound absorption [6,7]. However, the fabrication of very thin inclined solid strips separating individual straight slits is difficult, since the solid strips tend to deform and stick together. Another defect arising during the manufacture of all microstructures is internal surface roughness. The differences between the measured and predicted acoustic performance of 3D printed samples have been attributed to such imperfections introduced during the manufacturing process [2,8,9,10,11,12]. Direct numerical simulation of the influence of roughness in a relatively complicated microstructure using accurate knowledge of the roughness geometry has been found to be computationally expensive and an indirect modeling strategy involving a modified or effective dynamic viscosity coefficient of air. Although it is less computationally expensive than other methods, it does not predict the observed difference in the peak frequencies in the absorption spectra [10]. Manufacturing defects can affect absorption adversely. This has been found to be the case with stacked micro-slit layers [11]. However, this paper investigates the extent to which pore-wall roughness might improve the normal incidence absorption of a hard-backed layer containing identical slits.

Cylindrical samples containing 0.3 mm wide, parallel, and identical slits separated by 0.4 mm walls in a rigid resin matrix that are either vertical or inclined in zigzag patterns have been manufactured by 3D printing and their normal incidence sound absorption spectra measured in an impedance tube [12]. The zigzags overcome the difficulty that a significant proportion of parallel inclined slits in samples placed in an impedance tube would not be accessible to incident sound waves. Comparisons between measurements and predictions reveal discrepancies, especially for samples made by Fused Deposition Modeling (FDM).

The flow resistivity of the samples is sufficiently low that their sound absorption spectra are dominated by quarter-wavelength layer resonances. Between these resonances, the measured absorption is higher than predicted. Also, the second-order resonance in the measured absorption spectrum for the sample with vertical slits is at a lower frequency than predicted. It has been suggested that these discrepancies may be attributed to the rough internal surfaces and the uneven slit cross-sections perpendicular to the printing direction that occur during manufacturing [12]. For a given mean width, the flow resistivity in microchannels with wavy walls is predicted to be greater than if their width is constant [13]. In close-packed cubic arrays of insulating spheres and in media containing pores with fractally rough walls, the formation factor, which has the same influence on electrical conductivity as tortuosity has on acoustic wave propagation, is predicted to have relatively high values [14].

This paper outlines theories for the influence of two idealized forms of pore-wall roughness on flow resistivity and tortuosity and uses them to confirm that the inclusion of pore-wall roughness effects would improve agreement between data and predictions of the normal incidence absorption coefficient of the 3D printed slit pore samples. Since pore-wall roughness is found to increase sound absorption, the theories outlined are used also to explore the benefits of deliberately creating slits with periodic wall roughness.

Section 2 outlines theories of sound absorption by a hard-backed rigid solid layer containing parallel identical slits with cross-sections that either vary sinusoidally or have periodically alternating widths. In Section 3, after describing the manufacture of the samples for which normal incidence absorption spectra have been measured, these theories are used to confirm that pore-wall roughness is responsible for at least part of the observed differences between predictions and data for these 3D printed samples. In Section 4, the theories are used further to illustrate the extent to which varying slit cross-sections could improve the absorption of hard-backed rigid solid layers containing identical parallel slits. Some concluding remarks are presented in Section 5.

## 2. Theories of Slits with Regularly Varying Cross-Sections

### 2.1. Sinusoidal Slit Walls

**Figure 1:**
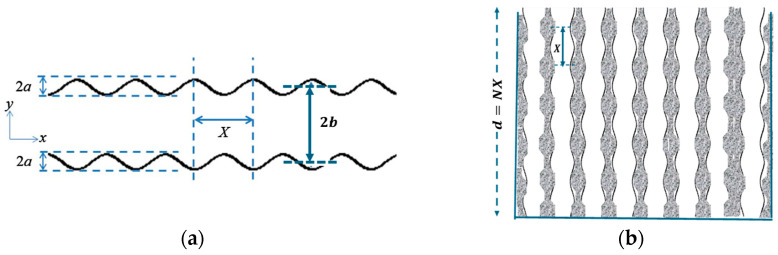
(**a**) A slit of mean width 2b and cross-section varying sinusoidally with amplitude a and wavelength X. (**b**) A porous hard-backed layer of thickness d containing vertical identical slits, each of which has the sinusoidal cross-section of Figure 1a, which extends for N wavelengths (d=NX).

Sound absorption by a rigid solid matrix containing parallel, identical, and inclined slits with sinusoidal cross-sections can be predicted analytically by extending a theory developed for microperforated plates [15]. Figure 1a shows a single slit with sinusoidal walls and a mean width of 2b. The sinusoidal variation in width has amplitude a and wavelength X. Figure 1b shows an array of such slits arranged vertically in a solid matrix to form a hard-backed porous layer with thickness d, which encompasses N wavelengths of the sinusoidal cross-section, so that X=d/N. Where the slits intersect the layer surface, their width is 2b. So, if the edge-to-edge spacing is 2w, the symmetry of the sinusoidal cross-sections means that the porosity at the surface $b/\left(b+w\right)$ is the same as the bulk porosity of a medium containing slits with a constant width of 2b.

If a/b≪1 and X≫a, the tortuosity $T_{r}$ of a slit of mean semi-width b with a cross-section that is sinusoidal with amplitude a and wavelength X is given by [10].

(1) $$T_{r}\left(a,X\right)=1+\left[\frac{e^{\frac{4\pi b}{X}}-1}{e^{\frac{4\pi b}{X}}+1}\right]\left(\frac{\pi a^{2}}{Xb}\right).$$

The flow resistivity of a uniform slit of semi-width b is $\left(\frac{3\mu }{b^{2}}\right)$, where μ is the dynamic viscosity of air [3]. With the sinusoidal cross-section shown in Figure 1a, the flow resistivity of the slit is increased by a factor S, which is given by [15].

(2) $$S\left(a,b,X\right)=\left\{\left[\frac{\left(\frac{1}{2}{\left(\frac{a}{b}\right)}^{2}+1\right)}{{\left(1-{\left(\frac{a}{b}\right)}^{2}\right)}^{2.5}}-\frac{1}{{\left(1-\frac{a}{b}\right)}^{3}}\right]\left[\frac{2e^{-\frac{4b}{5X}}}{1+e^{-\frac{4b}{5X}}}\right]+\frac{1}{{\left(1-\frac{a}{b}\right)}^{3}}\right\}.$$

If the slits are inclined at an angle θ to the surface normal, then the tortuosity and flow resistivity are increased by a further factor $T_{\theta }$ given by [7].

(3) $$T_{\theta }\left(\theta \right)=1/{\left(\cos\theta \right)}^{2}.$$

The flow resistivity of a medium with bulk porosity Ω and containing identical, inclined, and sinusoidal cross-section slits is given by [7,15].

(4) $$R_{S}\left(a,b,X.\theta \right)=\left(\frac{3\mu }{{\Omega b}^{2}}\right)T_{r}\left(a,X\right)T_{\theta }\left(\theta \right)S(a,b,X)$$

According to Stinson [16], the complex density and complex compressibility of air in a parallel-sided slit of width 2b, are written, respectively, as follows:

(5a) $$\rho \left(\lambda \right)=\rho_{0}/H\left(\lambda \right),$$

(5b) $$H\left(\lambda \right)=1-\tanh\left[\lambda \sqrt{\left(-i\right)}\right]/\lambda \sqrt{\left(-i\right)},$$

(5c) $$\lambda =b\sqrt{\omega /\nu },$$

(6) $$C\left(\lambda \right)={\left(\gamma P_{0}\right)}^{-1}\left[\gamma -\left(\gamma -1\right)H\left(\lambda \sqrt{N_{Pr}}\right)\right]\;\;.$$

where time dependence $e^{-i\omega t}$ is understood, $i=\sqrt{\left(-1\right)}$, ω is the angular frequency, ${\left(\gamma P_{0}\right)}^{-1}={\left(\rho_{0}{c_{0}}^{2}\right)}^{-1}$ is the adiabatic compressibility of air, γ, $P_{0}$, $\rho_{0}$, $c_{0}$, and $N_{pr}$ denote the specific heat ratio, atmospheric pressure, density, adiabatic sound speed, and Prandtl number for air, respectively, and $\nu =\mu /\rho_{0}$, with μ being the dynamic coefficient of viscosity.

The bulk complex density ($\rho_{b}\left(\lambda \right)$) and complex compressibility ($C_{b}\left(\lambda \right)$) for a medium of porosity Ω containing identical, parallel, and inclined slits with sinusoidal cross-sections are calculated from those for individual slits using Equations (1), (4), and (7a,b).

(7a) $$\rho_{b}\left(\lambda \right)=\left(T_{r}T_{\theta }/\Omega \right)\rho \left(\lambda \right),\;$$

(7b) $$C_{b}\left(\lambda \right)=\Omega C\left(\lambda \right).$$

### 2.2. Periodic Sectionally Uniform Slits

**Figure 2:**
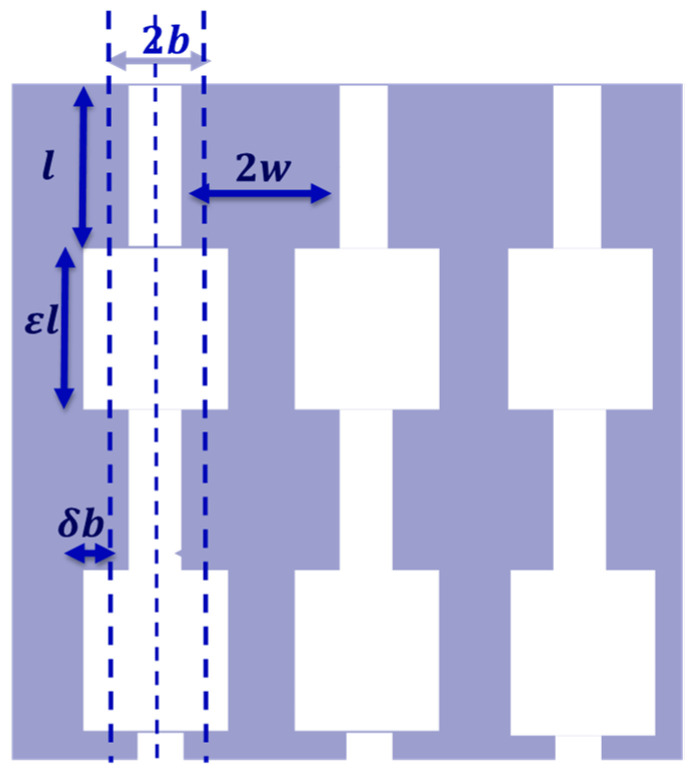
A solid matrix containing identical vertical sectionally uniform slits that have alternating widths of 2(b−δ) and 2b(1+δ) and a repeating cell length of $l\left(1+\varepsilon \right)$.

Consider a medium with vertical slits, which have alternating sections of length l and length εl with corresponding widths of 2(b−δ) and width 2b(1+δ), respectively, as shown in Figure 2. If the narrower sections of the slits intersect the surface, and their edge-to-edge spacing is 2w, the number of slits per unit surface area $n=\frac{1}{2b\left(1-\delta \right)+2w}$. The repeating structure or unit cell in each slit is composed of adjacent narrow and wide slit sections of total length $l\left(1+\varepsilon \right)$, so the number, N, of such cells per unit thickness of the layer is given by $N=\frac{1}{l\left(1+\varepsilon \right)}$.

Champoux and Stinson [17] have derived a general theory of the acoustic properties of a medium containing sectionally uniform pores of arbitrary shape and obtained results for alternating sectionally uniform cylindrical pores with different radii. Equations (18)–(20) in [17] can be used to derive analytical expressions for the porosity, flow resistivity, and tortuosity of a medium containing slits with regularly varying widths. 

Accordingly, the porosity is given by

(8) $$\Omega \left(\varepsilon ,\delta \right)=\left(\frac{b}{1+\varepsilon }\right)\left[\frac{1-\delta +\left(1+\delta \right)\varepsilon }{b\left(1-\delta \right)+w}\right].$$

The flow resistivity is given by

(9) $$\sigma \left(b,\varepsilon ,\delta \right)=\left(\frac{3\mu }{b^{3}}\right)\left[\frac{\left(b(1-\delta )+w\right)}{\left(1+\varepsilon \right)}\right]\left[\frac{1}{{\left(1-\delta \right)}^{3}}+\frac{\varepsilon }{{\left(1+\delta \right)}^{3}}\right].$$

The tortuosity is given by

(10) $$T_{R}\left(\varepsilon ,\delta \right)=\frac{\left[1-\delta +\varepsilon \left(1+\delta \right)\right]\left[\varepsilon \left(1+\delta \right)+1+\delta \right]}{\left[{\left(1+\varepsilon \right)}^{2}\left(1-\delta^{2}\right)\right]}.$$

The bulk dynamic density of the medium containing slits with periodically varying widths of the form shown in Figure 2 and inclined at θ to the normal to the surface is given by [17] (Equation (24)).

(11) $$\rho_{b}\left(\omega \right)=\left[\frac{\left(1+\delta \right)\rho \left(\lambda_{1}\right)+\varepsilon \left(1+\delta \right)\rho \left(\lambda_{2}\right)}{\left(1+\delta \right)+\varepsilon \left(1-\delta \right)}\right]\frac{T_{R}T_{\theta }}{\Omega },$$

where $\rho \left(\lambda_{1}\right)$ and $\rho \left(\lambda_{2}\right)$ are given by Equations (5a,b,c) after replacing b by $b\left(1-\delta \right)$ and $b\left(1+\delta \right),$ respectively, and $T_{\theta }$ is given by Equation (3).

Similarly, the bulk compressibility of the medium is given by [17] (Equation (29)).

(12) $$C_{b}\left(\omega \right)=\Omega \frac{C\left(\lambda_{1}\right)\left(1-\delta \right)+\varepsilon C\left(\lambda_{2}\right)\left(1+\delta \right)}{1-\delta +\varepsilon \left(1+\delta \right)}.$$

where $C\left(\lambda_{1}\right)$ and $C\left(\lambda_{2}\right)$ are given by Equation (6), with b replaced by $b\left(1-\delta \right)$ and $b\left(1+\delta \right),$ respectively.

Note that, for this regular variation in slit width, the acoustic properties depend on the proportion ε of wider to narrower cross-sections and the ratio δ of the widths, but as long as these lengths are small compared to the incident wavelength, they do not depend on the individual lengths of the sections with different widths. Also, it should be noted that the theory ignores the complicated flow velocity fields that may occur near the changes in width [6,17].

The complex wavenumber ($k\left(\omega \right)$) and relative characteristic impedance ($Z_{C}\left(\omega \right)$) of a porous material in which the pores are either slits with sinusoidal cross-sections of mean width 2b or periodically varying uniform widths with mean width 2b and repetition length $l\left(1+\varepsilon \right)$ are calculated, respectively, from Equations (7a,b) and (13a,b) or Equations (11)–(13a,b).

(13a) $$k\left(\omega \right)=\omega \sqrt{\rho_{b}\left(\omega \right)C_{b}\left(\omega \right)},$$

(13b) $$Z_{C}\left(\omega \right)={\left(\rho_{0}c_{0}\right)}^{-1}\sqrt{\rho_{b}\left(\omega \right)/C_{b}\left(\omega \right)}$$

The surface impedance of a hard-backed porous layer of thickness *d* is as follows:

(14) $$Z\left(d\right)=Z_{C}\left(\omega \right)coth\left(-ik\left(\omega \right)d\right)$$

The plane wave reflection coefficient, R(d), and normal incidence absorption coefficient, α(d), for a hard-backed porous layer are given by Equations (15a,b), respectively:

(15a) $$R\left(d\right)=\frac{\rho_{0}c_{0}-Z\left(d\right)}{\rho_{0}c_{0}+Z\left(d\right)},$$

(15b) $$\alpha \left(d\right)=1-\left|{\left(R\left(d\right)\right)}^{2}\right|.$$

## 3. Absorption by 3D Printed Samples

Cylindrical samples of diameter 29 mm and thickness 49.5 mm with vertical and zigzag slits have been created virtually in Computer Aided Design software (FreeCAD freeware version 0.19.2, https://www.freecadweb.org/) and manufactured using either a low-cost Fused Deposition Manufacturing (FDM) machine (FlashForge Creator Pro (Zhejiang Flashforge 3D Technology Co., Ltd., Hangzhou, China, EU store now NTI Sweden, AB) equipped with a 0.4 mm diameter nozzle, a heated bed (90 °C), and a layer resolution of between 100 and 500 microns) or a photopolymerization (LCD) device (Zortrax Inkspire, Zortrax, 10-410 Olsztyn, Poland), using an acrylonitrile butadiene styrene filament, a low-viscosity polymer, and photocurable resins (Zortrax Basic Pigment-free and Zortrax Pro Black) [12]. The FDM process consists of melting the polymer at 230 °C and extruding a continuous material layer-by-layer to build a final object. The samples with zigzag slits were printed on the more advanced LCD device, in which a thin layer of liquid resin is exposed to the UV light emitted from a liquid-crystal display and selectively cured on a platform to form the designed shape. The working platform is repeatedly lifted along the vertical direction by a single component layer thickness, and a new cross-section pattern is cured until the whole object is complete. Due to differences in the production processes, the LCD samples were created with a component layer thickness of 0.025 mm, compared to the layer thickness of 0.08 mm used in manufacturing the samples by FDM. To ensure the rigidity and proper spacing of the slit-separating walls, at least at the sample edges, ten equally spaced 0.4 mm wide and 1 mm thick clamping rings were created around the circumference of each sample [12].

Data obtained from impedance tube measurements on these samples [12] may be used to illustrate the potential usefulness of the theories developed in Section 3. The data are of the normal incidence absorption spectra for 49.5 mm thick samples containing 0.3 mm wide parallel slits, which are either vertical or with zigzags inclined at 45° to the surface normal. Nominally, the edge-to-edge slit separation at the surface of 0.4 mm implies a porosity of 0.429. Predictions obtained both analytically and numerically, the latter by using a finite element code, agree with each other but depart from the data [12]. Between the quarter-wavelength resonances, the measured absorption is higher than predicted. Also, for the vertical slits, the frequency of the second quarter-wavelength resonance in the measured spectrum is slightly lower than predicted. These discrepancies would be consistent with the samples having higher flow resistivity and tortuosity than predicted for slits with uniform cross-sections.

Microscope images of a cylindrical sample [12] indicate that the printed slit widths vary between 0.285 mm and 0.308 mm, i.e., between +5% and −2.7% of the nominal 0.03 m width. The thickness of the separating walls varies between 0.401 and 0.436 mm, i.e., by up to 9% of the nominal wall thickness of 0.04 m. It is suggested that these variations and other internal surface imperfections may have caused the discrepancies between predictions and data [12]. Although the variations in slit and wall widths in the 3D printed samples are neither sinusoidal nor sectionally uniform, it is interesting to investigate whether allowing for idealized forms of wall roughness can reduce the discrepancy between data and predictions.

**Figure 3:**
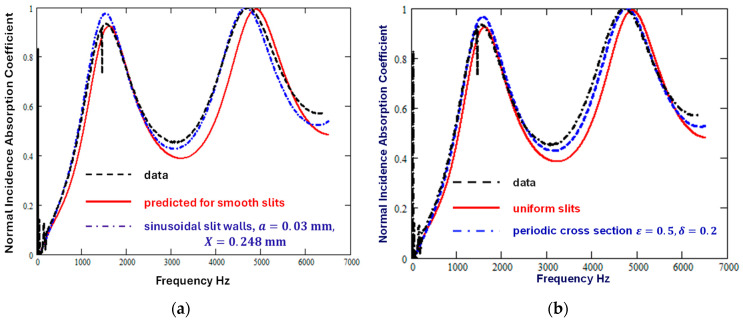
Comparison between the measured absorption spectrum of a 3D printed sample containing vertical slits (broken black line) [12] and predictions for vertical slits (solid red line) (**a**) with sinusoidal walls (a=0.03 mm, X=0.248 mm, dash–dot blue line) and (**b**) with a periodic variation in width (see Figure 2) with ε=0.5 and δ=0.2.

Figure 3a shows that the differences between predictions for uniform slits and data are reduced by assuming that the slit widths vary sinusoidally (Equations (1)–(10) with a=0.03 mm (i.e., 10% of the mean width) and X=0.248 mm (=d/200)). The corresponding values of $T_{r}$ and S are 1.076 and 1.325, respectively. Figure 3b shows that a similar reduction in the discrepancy between data and predictions for vertical uniform slits results from allowing for periodically uniform slit cross-section variation of the form shown in Figure 2 with ε=0.5 and δ=0.2.

To allow for the folds of length $L_{F}$ in the 3D printed samples with zigzag slits, it was found that an empirical tortuosity correction factor, $C_{F}$, given by

(16) $$C_{F}=\frac{4000L_{F}+{\left(\cos\theta \right)}^{2}}{4000L_{F}+1},$$

improved the agreement between analytical predictions for infinitely long slits inclined at 45° to the surface normal and finite element predictions for zigzag slits^2^. However, neither of these predictions yields significantly better agreement with the data than the prediction for infinitely long inclined slits.

**Figure 4:**
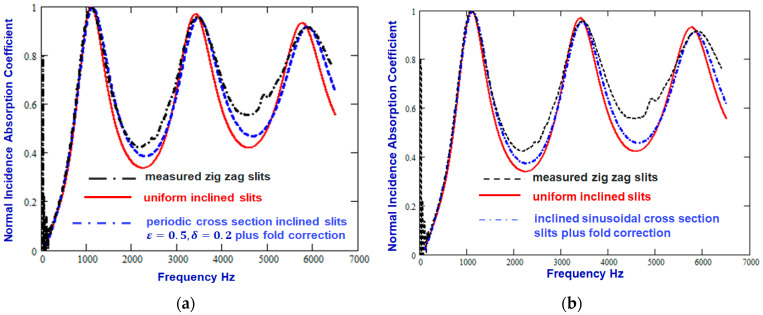
Comparison between the measured absorption spectrum of a 3D printed sample containing zigzag slits inclined at 45° to the surface normal (broken black line) [12] and predictions, including the fold correction, for infinitely long slits with parallel walls (solid red line) and (**a**) slits with sinusoidal walls (a=0.025 mm, X=0.248 mm, dash–dot blue line) or (**b**) slits with periodic variation in width (see Figure 2) with ε=0.5 and δ=0.2.

Figure 4a shows that the agreement with data of the prediction for infinitely long slits can be improved not only by allowing for folds in the slits with $L_{F}=1.25\;mm$ but also by assuming either that the slits have sinusoidally varying cross-sections with a=0.03 mm and X=0.248 mm, for which the corresponding values of $C_{F}$, $T_{r}$ and S are 0.92, 1.053, and 1.24, respectively. Figure 4b shows that there is a similar improvement in the agreement between data and the prediction for infinitely long slits after allowing for both the zigzag folds and a periodic variation in the slit cross-section of the form shown in Figure 2 with ε=0.5 and δ=0.2. Both comparisons suggest that slit wall roughness results in an increase in absorption between the layer resonances.

## 4. Increasing Absorption by Periodically Varying Slit Widths

**Figure 5:**
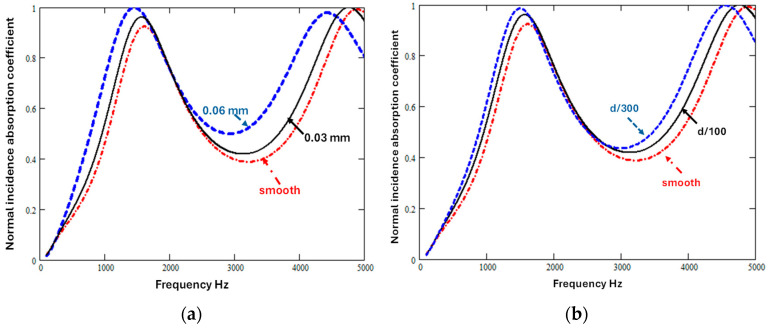
The predicted influence of sinusoidal slit parameters on normal incidence absorption coefficient of a 0.0495 m thick hard-backed layer containing vertical slits of nominal mean width 0.3 mm (**a**) due to varying the amplitude of the sinusoidal variation in slit cross-sections between 0 mm and 0.06 mm, assuming a constant wavelength of 0.248 mm, and (**b**) due to varying the sinusoidal wavelength between 0 mm and 0.165 mm, assuming a constant amplitude of 0.04 mm.

Equations (1)–(15) can be used to investigate the extent to which the deliberate introduction of sinusoidal variations in slit width or periodic variations in slit width might improve the absorption of a low flow resistivity sound absorber containing vertical slits with mean width 0.3 mm and porosity 0.429. Figure 5a shows the influence on predicted normal incidence absorption coefficient spectra as the sinusoidal variation amplitude is varied, while keeping the constant wavelength constant, and Figure 5b shows the influence of varying the wavelength at a constant amplitude.

Either increasing the amplitude for a given wavelength or decreasing the wavelength for a given amplitude lowers the first quarter-wavelength frequency and, because of the associated increases in tortuosity and flow resistivity, both changes increase the absorption between quarter-wavelength resonances. But, for a given wavelength, amplitude variation is predicted to have a greater influence.

**Figure 6:**
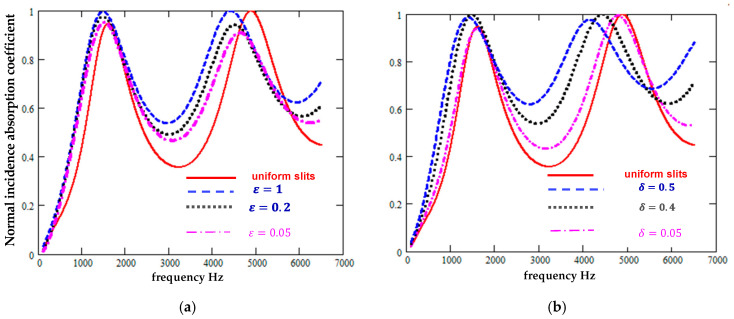
Predicted absorption spectra for a 0.0495 m thick hard-backed layer containing regularly repeating sectionally uniform slits normal to the surface of mean width 0.0003 m (**a**) as the wider width (δ = 0.2) fraction has values of 0, 0.05, 0.2, and 1 and (**b**) as the extra width of the wider parts (ε = 1) of the slits has values of 0%, 20%, 40%, and 50% of the mean widths.

Figure 6 suggests that periodically varying the slit widths also would improve the absorption of such a low flow resistivity hard-backed layer. As with the sinusoidal cross-sections, a regular sectionally uniform slit width variation is predicted to decrease the quarter-wavelength resonance frequencies as well as to increase absorption between these resonances. The width variation is predicted to have a greater influence than the proportion of slit sections that are wider.

As the change in the periodically varying slit width increases, the resulting slit microstructure begins to resemble that of a ‘pancake’ absorber in which each module consists of a central cylindrical main channel with dead-end side branches [18]. A hard-backed layer of such a microstructure has been found to achieve good low-frequency absorption, albeit with narrow layer resonance peaks in the normal incidence absorption spectrum. The main influence of this microstructure is on the complex compressibility [18].

**Figure 7:**
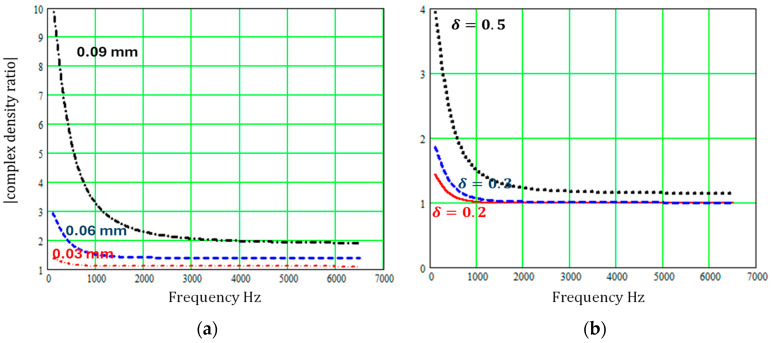
Predicted influence of regularly varying slit cross-sections on the modulus of the complex density relative to that for constant cross-section pores of width 0.3 mm (**a**) for sinusoidal slit walls with the wavelength fixed at 0.0248 mm and amplitudes of 0.03 mm, 0.06 mm, or 0.09 mm (**b**) for regular sectionally uniform slit width variations, with ε=1 and δ = 0.2, 0.3, or 0.5.

**Figure 8:**
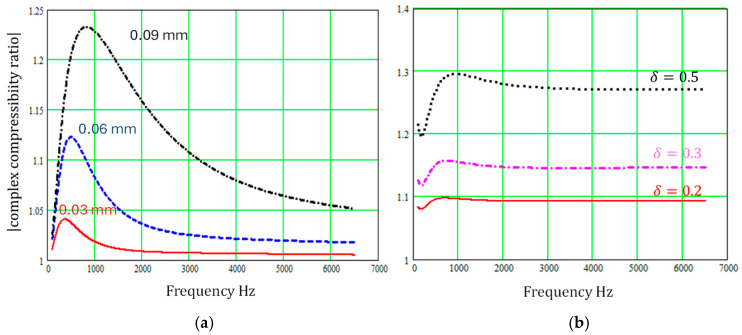
Predicted influence of regularly varying slit cross-sections on the modulus of the complex compressibility relative to that for constant cross-section pores of width 0.3 mm (**a**) for sinusoidal slit walls with the wavelength fixed at 0.0248 mm and amplitudes of 0.03 mm, 0.06 mm, or 0.09 mm (**b**) for regular sectionally uniform slit width variations, with ε=1 and δ = 0.2, 0.3, or 0.5.

Although the sinusoidal cross-section and regular repeating sectionally uniform slit microstructures may not create absorption peaks at as low frequencies as the ‘pancake’ absorber, Figure 7 and Figure 8 show that varying slit cross-sections have a large influence on both complex density and complex compressibility at low frequencies, and their influence persists over a wide frequency range.

It should be noted, however, that the theory presented for sinusoidal pore walls is increasingly inaccurate as a/b increases, and the assumptions used in the theory of regularly varying sectionally uniform slits become less tenable as the slit width variation increases.

## 5. Conclusions

Theories for the influence of sinusoidal variations and of regular uniform variations in slit widths have been outlined and used to confirm that at least part of the discrepancies between measured and predicted normal incidence absorption spectra for 3D printed samples with vertical and zigzag slits [12] can be attributed to variation in the width of the slits in the manufactured samples. The presence of wall roughness increases the absorption between the layer resonance peaks and decreases the frequency of the quarter-wavelength layer resonance because of increased tortuosity and flow resistivity.

Furthermore, the extent to which the deliberate introduction of either a sinusoidal variation or a sectionally uniform variation in slit width can improve the absorption offered by a rigid frame absorber containing vertical slits with constant widths has been investigated. The amplitude of the sinusoidal variation for a given wavelength has a larger influence on the predicted normal incidence absorption spectrum than the variation in wavelength for a given amplitude. Similarly, the ratio of different width section lengths for a given ratio of lengths of different width sections has a larger influence on the predicted normal incidence absorption spectrum than the ratio of lengths of different width sections, for a given width ratio.

Periodically varying slit widths are predicted to influence both the complex density and complex compressibility of a slit pore medium over a wide frequency range.

Although, with current 3D printing technology and a relatively simple microstructure of identical vertical or slanted slits, it is difficult to provide broadband sound absorption, the wider-band and lower-frequency absorption associated with more elaborate slit-based micro- and macro-structures [6,19,20] should be improved by introducing periodic variations in slit width, thereby enhancing tortuosity and flow resistivity.

## Data Availability

The original contributions presented in this study are included in the article. Further inquiries can be directed to the corresponding author.

## References

[1] Fotsing E.R., Dubourg A., Ross A., Mardjono J. (2019). Acoustic properties of periodic micro-structures obtained by additive manufacturing. Appl. Acoust..

[2] Boulvert J., Costa-Baptista J., Cavalieri T., Perna M., Fotsing E.R., Romero-García V., Gabard G., Ross A., Mardjono J., Groby J.-P. (2020). Acoustic modeling of micro-lattices obtained by additive manufacturing. Appl. Acoust..

[3] Guild M.D., Garcia-Chocano V.M., Kan W., Sanchez-Dehesa J. (2015). Acoustic metamaterial absorbers based on multilayered sonic crystals. J. Appl. Phys..

[4] Nori M., Venegas R. (2017). Sound propagation in porous materials with annular pores. J. Acoust. Soc. Am..

[5] Tang Y., Ren S., Meng H., Xin F., Huang L., Chen T., Zhang C., Lu T.J. (2017). Hybrid acoustic metamaterial as super absorber for broadband low-frequency sound. Scient. Rep..

[6] Ren S.W., Meng H., Xin F.X., Lu T.J. (2016). Ultrathin multi-slit metamaterial as excellent sound absorber: Influence of micro-structure. J. Appl. Phys..

[7] Attenborough K. (2018). Microstructures for lowering the quarter wavelength resonance frequency of a hard-backed rigid-porous layer. Appl. Acoust..

[8] Ciochon A., Kennedy J., Leiba R., Flanagan L., Culleton M. (2023). The impact of surface roughness on an additively manufactured acoustic material: An experimental and numerical investigation. J. Sound Vib..

[9] Kennedy J., Flanagan L., Dowling L., Bennett G.J., Rice H., Trimble D. (2019). The Influence of Additive Manufacturing Processes on the Performance of a Periodic Acoustic Metamaterial. Int. J. Polym. Sci..

[10] Ciochon A., Kennedy J. (2024). Efficient modelling of surface roughness effects in additively manufactured materials. Appl. Acoust..

[11] Xiang N., Hoeft M., Fackler C.J., Chen Z., Barach P. (2023). Validation of Bayesian design for broadband microslit panel absorbers using causal inference. J. Acoust. Soc. Am..

[12] Opiela K.C., Zielinski T., Attenborough K. (2022). Limitations on validating slitted sound absorber designs through budget additive manufacturing. J. Mater. Des..

[13] Wang H., Wang Y. (2007). Flow in microchannels with rough walls: Flow pattern and pressure drop. J. Micromech. Microeng..

[14] Schwartz L.M., Sen P.N., Johnson D.L. (1989). Influence of rough surfaces on electrolytic conduction in porous media. Phys. Rev. B.

[15] Song S.Y., Yang X.H., Xin F.X., Ren S.W., Lu T.J. (2017). Modeling of roughness effects on acoustic properties of micro-slits. J. Appl. Phys. D.

[16] Stinson M.R. (1991). The propagation of plane sound waves in narrow and wide circular tubes, and generalization to uniform tubes of arbitrary cross-sectional shape. J. Acoust. Soc. Am..

[17] Champoux Y., Stinson M. (1992). On acoustical models for sound propagation in rigid frame porous materials and the influence of shape factors. J. Acoust. Soc. Am..

[18] Dupont T., Leclaire P., Panneton R., Umnova O. (2018). A microstructure material design for low frequency sound absorption. Appl. Acoust..

[19] Attenborough K. (2019). Macro- and micro-structure designs for porous sound absorbers. Appl. Acoust..

[20] Zielinski T.G., Opiela K., Dauchez N., Boutin T., Galland M.-A., Attenborough K. (2024). Extremely tortuous sound absorbers with labyrinthine channels in non-porous and microporous solid skeletons. Appl. Acoust..

